# Developing an Older Adult Volunteer Program in a New York Chinese Community: An Evidence-Based Approach

**DOI:** 10.1007/s12126-012-9160-5

**Published:** 2012-09-29

**Authors:** Ada C. Mui, Myra Glajchen, Huajuan Chen, Juanjuan Sun

**Affiliations:** 1 Sau Po Centre on Ageing, University of Hong Kong, Hong Kong, China; 2 Columbia University School of Social Work, 1255 Amsterdam Ave, New York, NY 10027 USA; 3 Department of Pain Medicine and Palliative Care, Beth Israel Medical Center, First Avenue at 16th Street, New York, NY 10003 USA; 4 Brown School of Social Work, Washington University in St. Louis, One Brookings Drive, St. Louis, MO 63130 USA; 5 Institute of Gerontology, Renmin University of China, 59, Zhongguancun Street, Haidian, Beijing, China

**Keywords:** Older volunteers, Chinese immigrants, Friendly callers, Telephone assurance

## Abstract

This study reports the results of a pilot volunteer project for older Chinese immigrants and documents benefits for both volunteers and caregiver recipients. Using a social marketing approach, the volunteer project was designed as a social model to promote better health among older Chinese immigrants in New York City. The packaging of this health promotion project as a volunteer program was based on a strengths perspective. In the program, 18 older Chinese immigrants were trained to provide support and referral to family caregivers of ill relatives in the Chinese community. At 6 months, outcomes were evaluated for both volunteers and caregivers. The older volunteers perceived benefits associated with volunteering, specifically, a greater sense of well-being and satisfaction with life. In addition, the majority of volunteers felt empowered by training and volunteering (100 %), felt the skills they learned improved communication with their own families (90 %), and reported physical and emotional health benefits (61 %). At the same time, caregivers reported stress reduction following volunteer support. Findings suggest that a volunteer program model may be an effective health promotion intervention for older Chinese immigrants.

Globally, the proportion of people aged 60 and over is growing faster than any other age group as a result of longer life expectancy and a decline in fertility rates (United Nations 2002). Today, one in ten people in the world is aged 60 or older, and it is projected that the older population in the world will quadruple, growing from about 600 million to almost 2 billion by 2050, when one out of five people will be aged 60 or older (United Nations 2002). This global aging phenomenon is no more evident anywhere in the world than in China.

By 2020, China’s older population aged 60 and over is estimated to reach 248 million. By 2051, China’s aging population is expected to peak at 438 million, which, in all likelihood, will outnumber the entire US population, which is estimated at around 400 million using middle series projections (US Census Bureau 2004). Older people can expect to live and stay productive for at least 20 to 30 years after retirement; population aging, therefore, is a rich stock of human capital in our global society (Morrow-Howell et al. 2001). This human capital represents a person’s productive capacity in terms of knowledge and skills, physical and mental health conditions, life experience and positive attitude (Wei 2007). This demographic evidence of rising longevity is happening around the world in almost every country. With a record-setting wave of baby boomers now reaching retirement age, there will be an increasing number of older people in each community who can be mobilized to engage in productive community services. This growing aging population is much more educated than their previous cohorts and is exceptionally skilled and experienced (Mui and Shibusawa 2008; Zedlewski and Butrica 2007). In addition, the social networks garnered over the lifetime of an older person can be an asset of tremendous social capital for communities and countries. More importantly, public health research has established that productive role engagement in later life generates health and mental health improvement for older people (Glass et al. 2004; Morrow-Howell et al. 2009; Zedlewski and Butrica 2007).

Productive engagement through formal volunteering in an organization, caregiving, life-long learning, and employment, is documented to have significant beneficial effects on older adults’ health and psychological well-being (Mjelde-Mossey et al. 2007; Morrow-Howell et al. 2009). Specifically, research indicates that engaging in formal volunteering and work benefits not only the older volunteers but also the recipients of volunteer services, the organizations that count on them, and the economy (Zedlewski and Butrica 2007). In the 2004 U.S. Health and Retirement Study, more than 50 % of older people under age 75 indicated their interest in both paid work and volunteer opportunities. Although older adults desire to be involved in meaningful roles and stay active, the availability of productive roles has lagged.

This paper describes how theory and research guided the development, implementation, and evaluation of a pilot volunteer program for older Chinese immigrants in New York City entitled the Phone Angel Program. “Phone Angels” were older Chinese-American volunteers trained to provide telephone assurance, emotional support, and referrals to Chinese American caregivers of sick relatives. Most of the Phone Angels and caregivers were first generation immigrants who came to the US at an older age and preferred to communicate in their native language; the Chinese cultural and linguistic affinity was an asset in the helping process. The Phone Angel Program was effective in creating meaningful new roles for older Chinese volunteers and opportunities for them to meet the critical needs of Chinese caregivers isolated due to language barriers and caregiver burden.

## Volunteerism in Later Life

One third of the human lifespan will be spent in post-retirement; this period has been coined the “Third Age” in gerontology literature (Cornman 1997; Mui 2010). Living longer should be considered a new and beneficial opportunity for older individuals, families, and communities. This new outlook and the potential value of this human capital is extremely important for the baby boom generation, as they are more likely to be educated, skilled, resourceful, and more willing to be socially engaged than their parents’ cohort (Mui 2010; Mui and Shibusawa 2008). Research has documented the health and psychological benefits of productive engagement in later life. Specifically, studies show positive associations between volunteer engagement and reduced morbidity and depression, improved health and strength, greater happiness, and enhanced cognitive ability (Greenfield and Marks 2004; Li and Ferraro 2005; Musick and Wilson 2003; Willigen 2000). Possible explanations for the association between formal volunteering and improved health and mental health outcomes may include increased intellectual stimulation, mental activity, on-going meaningful social interactions with fellow volunteers, social support, and a sense of purpose in volunteering roles (Kubzansky et al. 2000; Mui and Shibusawa 2008). There is also enhanced self-image and social status as a volunteer, and greater access to social, psychological, and material resources that can be empowering and fulfilling (Morrow-Howell et al. 2009; Thoits and Hewitt 2001; Wilson 2000). Training and learning associated with volunteer work may encourage older adults to develop more knowledge and skills that boost their self-efficacy, intellectual capacity, and motivation to learn (Greenfield and Marks 2004; Harlow-Rosentraub et al. 2006). Finally, older volunteers show improved physical and mental health and often gain personal fulfillment and a sense of purpose when helping people in need.

Volunteerism in the US has been established for several decades among older people. Two important federal programs that promote volunteerism in older people were started in the 1960s: the Senior Companionship Program, which provides financial support to low-income adults 60 years and older who serve as home care assistants to needy older persons; and the Retired and Senior Volunteer Program (RSVP), which supports and trains older volunteers to serve their communities. National Senior Corps programs in the US have played an important role in promoting volunteering among old people—approximately half a million older adults have volunteered in their programs—as have a number of other organizations such as Executive Service Corps and the National Retiree Volunteer Coalition.

Research on and evaluation of older volunteers programs suggest that they could meet critical needs for their communities. For example, “Experience Corps” provides evidence that older volunteers have the capacity to tutor children with learning difficulties and improve their academic performance (Gattis et al. 2010). The evaluation of the “Family Friends Program” found that older volunteers who provided home visit services significantly reduced hospitalization rates among chronically ill children and improved the well-being of family caregivers (Rinck and Naragon 1995). In addition, teens with behavioral problems and school challenges also benefited from older mentors in the “Across Ages Program.” The teens who participated showed improved class attendance, better attitudes toward school, and a reduced rate of substance use (Rogers and Taylor 1997). Wheeler et al. (1998) reviewed 37 program models and found that 85 % of the individuals served by older volunteers showed significantly better outcomes when compared with service recipients of different age groups (cited by Zedlewski and Butrica 2007). These older adult volunteer programs demonstrate that the “elderly” are not a group of infirm people, but are important human capital capable of addressing difficult social problems in their communities.

Formal volunteer activities by older adults benefit the economy as well. Since 2002, older Americans contributed an estimated US$161.7 billion to society per year through provision of unpaid services. Out of this total amount, 28 % was from volunteering in formal programs, 61 % was from caregiving support provided to family and grandchildren, and 11 % was through informal volunteering to help people and communities in need. Older population’s contributions in these areas lessen demand for public services provided at government cost, leaving the government with resources that could be used for other social programs. From a cost-benefit perspective, the older volunteers’ human capital in terms of intelligence, skills, and experience provides monetary and social value that exceeds the investment required to manage and train them (Zedlewski and Butrica 2007).

## Conceptual Frameworks Underlying the Phone Angel Program

### Strengths, Empowerment, and Social Capital Perspectives

Recent landmark research on Asian American experience that used regional probability sampling suggests that 46 % of the older Chinese-American immigrant population in New York City is depressed (Mui and Kang 2006; Mui and Shibusawa 2008). Regression analyses document that predictors of higher level depression in this population are poor health, high levels of stress, dissatisfaction with family, perceived generation gaps, lack of social support, and feelings of social isolation (Mui and Kang 2006). These findings suggest that provision of health promotion, family communication skills, training in problem solving strategies, stress management, and enhancement of social support and social connection through productive engagement may be effective in maintaining mental health for older Chinese-American adults. From a strengths perspective, that is, older Chinese-American immigrants are resilient and capable of productivity, if society can provide education and training to empower them. Empowerment is the process of social interaction of individuals and groups that intend to enable people to enhance their individual and collective skills to participate in a community (Erben et al. 1999). The focus on strengths and empowerment perspective is characterized by its positive and optimistic view of people confronted by life’s challenges.

The Phone Angel Program was conceptualized as a social model for health promotion targeting older Chinese immigrants. The Program aimed to improve mental health and self-efficacy of older ethnic Chinese immigrants through a program designed to empower and educate older Chinese-American volunteers while simultaneously providing emotional support to caregivers of ill relatives within the Chinese community. The program content and training in family dynamics, caregiving burden, communication and problem-solving, stress management, and health education had a dual focus: preparing volunteers for their roles as Phone Angels while also empowering volunteers themselves so they could apply their new skills in family communication, problem solving, and self-help to their own life situations.

Social capital theory also guided the conceptualization of the Phone Angel Program because social capital emphasizes the quality of social relations and their impact on the lives of the participants (Erben et al. 1999). Participants in the Phone Angel Program needed to demonstrate trust and confidence in each other. This mutually supportive relationship helped them as a social group to become successful in their volunteering mission (Coleman 1990). In the Phone Angel Program, we encouraged bonding and shared learning at the inception of the program, during the intensive training month, and then during the ongoing phase of training, sharing, and supervision. Because social capital also refers to sociability and social status of the individual within the group, the opportunity to adopt the new volunteer role and the title “Phone Angel” gave the older Chinese volunteers a meaningful and admirable social status in the senior center and among their social network of friends and families. In addition, the collaboration among social workers in the senior center, the hospital-based principal investigator, the university social work researcher, and the funding agency was envisioned to generate a stock of social capital when applied beyond the single pilot program. Moreover, the collaboration among a team from different academic and social service systems resulted in the creation of a new infrastructure that can lead to future collaboration and sustainability of similar productive aging programs.

### Social Marketing Approach

From a public health perspective, the challenges of population aging and longevity point to the importance of developing health promotion programs to help prevent disease and maintain the health of the older population. In the U.S. context, efficacious and cost-effective strategies to promote health such as Experience Corps have been conceptualized as social marketing interventions—marketing public health through older volunteering activities (Fried et al. 2004; Glass et al. 2004; Tan et al. 2010). Experience Corps in particular has been described in medical literature as a social model for health promotion (Tan et al. 2010). However, these strategies have been used mostly for English-speaking older Americans. The Phone Angel Program was modeled after Experience Corps’ social marketing approach, and designed as a health promotion program for older Chinese-Americans in New York City.

The social marketing approach has been used to guide health promotion programs for more than two decades. Health promoters realized that the same marketing principles that were being used to sell products to consumers could be used to “sell” ideas, attitudes, and behaviors. Social marketing is different from other areas of marketing because the objective of the marketer is to influence social behaviors to benefit the target population and the community not the marketer (Kotler and Roberto 1989). Research indicates that older Chinese immigrants (our target group) were less likely to seek out social services or counseling for unmet needs and problems because of their lack of trust in the service delivery system (Mui and Shibusawa 2008). In addition, older Chinese persons were less likely to participate in volunteering and less willing to get involved in another family’s issue (Mui and Shibusawa 2008). This may have been due to the Chinese cultural norms of not interfering in another person’s life (“各家自掃門前雪” literally means family should only shovel snow in their own front yard). Culturally, older Chinese were less likely to acknowledge family problems for fear of “losing face” (Mui and Kang 2006). For all of these reasons, we decided that a social intervention that promotes health for older Chinese and aims at building trusting relationships among members of a Chinese community was worth testing.

The social work team decided to use the social marketing approach to sell the volunteer program, with the dual mission of health promotion and building trusting relationships among older Chinese immigrants. Social marketing presents a formula by which health programs can be packaged and transmitted to the “target population” (older Chinese immigrants). Social marketing is about packaging the program so that the desired target population will buy-in and adopt the program (product) for themselves (Kotler and Roberto 1989). Social marketing uses marketing principles to promote interventions that enhance desirable health and psychological well-being. Social marketing-based mental health interventions could be particularly useful in addressing depressive mood, where counseling alone may not be effective. Social marketing differs from traditional health education in that the health promotion outcome may not be identical to the product or behavior being promoted. The Phone Angel Program was conceptualized and marketed to the Chinese-American older adults in a community senior center with the hope that the knowledge and skills they obtained during the training sessions would empower them, help them to establish trusting relationships, promote social bonding, reinforce social support, and promote mental health. In addition, it was hoped that the Phone Angel Program could benefit immigrant Chinese elders who are underrepresented in traditional public health interventions, and therefore at higher risk for poor mental health.

## Methods

### Conceptualization and Design of the Phone Angel Program

The Phone Angel Program was conceptualized as a health promotion social intervention for older Chinese immigrants in New York City. The Program was also designed to address the caregiver burden in Chinese immigrant families who experience additional stresses of linguistic and social isolation. Many older Chinese-American families in New York City have very low English proficiency. Cultural and linguistic isolation can be compounded by illness, caregiving, and living circumstances in New York City’s Chinatown. The Phone Angel program was designed to train volunteers to serve as friendly volunteers for isolated caregivers and provide them emotional and coping skill support in their native language. The program was designed to empower both the caller and the recipient.

### Goals of the Phone Angel Program

The goals of the pilot Phone Angel Program at the individuals level were: (1) to increase volunteers’ trust, confidence, social engagement, and social support; (2) to increase volunteers’ knowledge and skills in active listening, problem-solving, family communication, and stress management; (3) to improve volunteers’ self-efficacy and sense of fulfillment in life; (4) to lower caregivers’ sense of loneliness and suffering from caregiving burden. The goals of the program at the organizational level were: (1) to evaluate the effectiveness of recruitment methods; (2) to evaluate the usefulness and appropriateness of the training curriculum; (3) to evaluate the effectiveness of the infrastructure of the social work team; (4) to evaluate the outcomes of the Phone Angel Program for both older volunteers and caregiver recipients.

### Implementation of Phone Angel Program

The Phone Angel Program involved recruitment, knowledge and skills training, consistent supervision, documentation of contact with caregiver and difficulties encountered, a support group to share volunteer experiences, sharing of personal issues among volunteers, and a program evaluation including an assessment of volunteer efficacy and caregiver stress before and after training and services.

The program was conceptualized during the summer of 2010 by a university social work researcher and a hospital-based social work researcher, then further developed with input from social workers in a senior center. The University partnership was critical because the social work researcher brought expertise in developing a knowledge-based training curriculum and scientific evaluation of the program. This expertise was complemented by the hospital-based researcher’s in-depth knowledge about caregiver burden and cultural burden among older Chinese Americans. The hospital-based researcher obtained financial support for the pilot, and the social work team agreed on the roles of each partner organization before implementation of the program in November 2010.

### Recruitment and Training

Volunteers were recruited in various ways. First, a bilingual flyer with an overview of the Phone Angel program was distributed at the senior center. The flyer included information about the Phone Angel Program, the stipend, the 6-month commitment, and the telephone support component. Seniors were invited to a general meeting to learn more about the program. The meeting, held at the beginning of November, was attended by 250 seniors, and 19 signed up to become volunteers. Immediately after the meeting, potential volunteers communicated with the social worker in the center to enquire about the program in greater detail. After people signed up, the social worker followed up with a call to each potential volunteer to confirm their participation and to perform a brief screening. The same 19 volunteers confirmed their interest, and all were deemed appropriate by the social worker. Chinese caregivers of ill relatives with unmet needs were identified by the hospital-based social worker and referred to the senior center social worker for matching with volunteers based on Chinese dialect and gender.

Training was comprised of 72-h, intensive training sessions between November 2010 and February 2011, followed by ongoing training sessions every 3 to 4 weeks. As most of the volunteers had no prior family caregiving experience, early training sessions provided context and knowledge about the plight of family caregivers, expectations for their role as volunteers, caregiver burden and life event stressors, active listening and communication skills, coping and problem-solving skills, and family dynamics. Volunteers were trained to provide telephone support to caregivers using Mandarin or Cantonese, whichever language the caregiver had the most linguistic comfort with, at least once a week. Volunteers were educated in basic empathy, learning to assess the needs of caregivers, not adding to the burden of caregivers by calling too frequently, and evaluating when would be the best time to call.

The following topics were covered:

1. Introduction to population aging issues.
2. The family caregivers’ needs and complex emotions.
3. Tasks and Role of Phone Angel volunteers.
4. Basic Communication Skills and Effective Listening.
5. Tips for suggested coping skills/problem solving skills
6. How do I talk to the caregiver when I call?
7. Special Tips for Me as a Volunteer Phone Angel: Do’s and Don’ts for volunteers.

Initially, training was conducted every 2 weeks, but as volunteers gained confidence and a sense of competence in their role as volunteers for caregivers, training was spaced to 3 or 4 weeks apart. Later training sessions were focused on sharing, supervision, and processing and problem-solving difficult caregiver situations. Between meetings, additional monitoring and support were available to the Phone Angels and caregivers from their respective social workers. Role plays were conducted during the training sessions to develop the volunteers’ communication skills. As volunteers showed readiness for their Phone Angel duties, they were assigned a caregiver.

### Monitoring and Evaluation

Ongoing training and support of the Phone Angel volunteers was fundamental to the success of the program. To establish this, it was necessary to develop a core infrastructure through which the social workers at the hospital and the senior center met regularly to coordinate the matching of the Phone Angels with identified caregivers and to discuss and resolve issues as they arose.

Phone Angels were each assigned to one, two, or three caregivers and instructed to call each caregiver at least once a week but up to three times a week as needed. Assignments were based on the Phone Angel’s primary language and gender. Caregivers received an average of three to eleven calls each before the completion of the program, depending on their level of need. Most Phone Angels provided emotional support, practical advice about community resources, guidance about family conflict, and advice about future care planning for the ill relative. In terms of boundaries, Phone Angels were advised to keep calls to 30–60 min in duration, to focus on the needs of the caregiver, and to maintain the relationship over the phone rather than suggesting face-to-face contact. Phone Angels were conscientious in keeping records and producing a weekly report to document the frequency of their calls, the content of each call, and the actions taken. Every call was carefully assessed in terms of the volunteers’ sensitivity and active listening skills. If volunteers encountered a complicated situation that required professional intervention, caregivers would be referred back to the hospital social worker for follow-up. Such cases included the need for ongoing or new medical care, questions about housing, and requests for more paid assistance in the home. Other issues included finding the optimal time for making calls and trying to engage a caregiver who was talking in front of the ill relative using speaker phone.

Phone Angel volunteers received a $50 stipend after the intensive training program and another $50 after 6 months of service. The stipend offset volunteers’ travel expenses. It was also a meaningful incentive for volunteers, not only because the majority of the Phone Angel Program volunteers were low-income, but also because payment gave them a sense of dignity and tangible societal worth to their families. The financial cost of maintaining the volunteer service was low because volunteers could call Chinese family caregivers from both the senior center and their own homes. Calling cards were provided to the Phone Angels so that they did not have to use their own phones, which protected their privacy.

## Results

Nineteen older Chinese seniors signed up as volunteers, one dropped out, and 18 completed the program. The mean age of volunteers was 72.1 (range: 64–86). Phone Angels were disproportionately women (72 %), married (89 %), and born in mainland China (94 %). In terms of perceived health, 72 % reported that their health condition was “fair,” 22 % reported “good” health, and only 6 % reported “excellent” health. Ninety-four percent of the Phone Angels felt that their English proficiency was fair to poor, and listed Chinese as their preferred language in daily interaction with family and friends. They were considered well educated in their home country (44 % with at least some high school education).

After 6 months of service, the social work researcher assessed perceived benefits of the Phone Angel Program for volunteers, using a focus group and a short questionnaire with closed and open-ended questions. Results show that the majority of the older volunteers perceived great benefits from their participation in the Phone Angel Program. These included a better sense of self, self-efficacy, and time management; better relationships and communication skills; higher levels of awareness about social issues; and a sense of improved mental health and emotional well-being

Using a survey instrument with closed-ended questions worded to attribute any perceived change due to the Phone Angel Program, volunteers were asked to rate or endorse the descriptive items listed below. Some statements used rating options of “Agree” or “Disagree,” while others used response options of “worse,” “same,” and “better.”

The following are the descriptive data on perceived benefits reported by the Phone Angel older volunteers. All items started with “After joining the Phone Angel Program”,

1. I feel empowered and happier because I have the opportunity to serve others (100 %)
2. I feel I have made a contribution to the community through this Program (78 %)
3. I have developed a stronger sense of purpose in my life (100 %)
4. I have developed an ability to improve personal time management skills (78 %)
5. I have developed a better understanding of my own emotions (83 %)
6. I have enlarged my social circle of friends (83 %)
7. I feel better about myself (67 %)
8. I have developed better communication skills (100 %)
9. I have developed a bigger vision about life (89 %)
10. I am better able to share the knowledge and skills I learned with my family and friends (89 %)
11. I feel that my family is supportive of me in doing volunteer work (72 %)
12. I know my family is very proud of me being a Phone Angel to help others (56 %)
13. My family is more aware of community issues (72 %)
14. My relationship with my family has improved (72 %)
15. I am considered a role model by my family (72 %)
16. My spouse and I have become more active in social activities (61 %)

At the end of the questionnaire, open-ended questions were used to elicit the perceived effects of Phone Angel Program participation on personal growth and family relationships, while also providing the opportunity to make “additional comments” to share perceptions about the impact of the volunteer experience on their lives. Volunteers were asked to rate whether the intensive training and on-going support were useful for them in fulfilling their volunteer responsibilities. Volunteers also reported their perception of the impact of their services to the caregivers. Eighty-nine percent of Phone Angels felt that their work improved caregivers’ lives and caregivers were very responsive and appreciative of their support. Two Phone Angels felt that caregivers did not respond to them initially and felt that their work was not appreciated as expected. However, they learned that these caregivers were too stressed to receive help and needed more time to establish rapport with the Phone Angels.

In terms of the usefulness of the training curriculum, 89 % of the Phone Angel volunteers felt that the curriculum, supervision, and on-going support were “extremely useful” for them and only 11 % felt they were just “useful.” Regarding perceived health, 61 % of the Phone Angels felt that their health was “better” as a result of participation in the program, and 39 % stated “same.” These findings suggest that these older Chinese volunteers in the Phone Angel program perceived benefits to themselves, to caregiver recipients, and to family. When asked about other effects associated with the Program participation, all volunteers reported that they learned to improve their time management skills by scheduling training sessions, meetings, and friendly calls to caregivers.

Twenty-eight caregivers who received support from Phone Angels were assessed using the Brief Assessment Scale for Caregivers (BASC) in Chinese as well as other measures specifically designed for the program. As a group, these caregivers felt that Phone Angel volunteers reduced their stress and burden, listened well, and made good suggestions when problems were presented. The impact of the program for caregivers is described in another manuscript (Glajchen and Mui 2011).

## Discussion and Conclusion

The primary goals of the Phone Angel volunteer program were two-fold: to empower and provide lifelong learning opportunities for older adult Chinese American volunteers and to benefit Chinese American caregivers in the community. Results from the pilot showed the potential for a volunteer program to meet both goals simultaneously. Older volunteers and caregiver-recipients reported positive outcomes from their participation in the program. These findings are consistent with the literature showing that older adults who are engaged in volunteer activities perceive positive psychosocial outcomes (Fried et al. 2004; Mjelde-Mossey et al. 2007; Morrow-Howell et al. 2009). From the descriptive data presented here, older Chinese volunteers felt empowered, and these benefits might be associated with increased self-esteem (having better sense of self and sense of purpose), improved self-efficacy (better communication and problem-solving skills), increased knowledge (caregiving experience, health information, time management, increased information and resources), and increased mental health and happiness (strengthened trusting relationships with other volunteers and caregivers as well as increased social bonding and social support among volunteers). These findings are consistent with the literature suggesting that volunteer engagement can be an effective social intervention for health promotion (Fried et al. 2004; Tan et al. 2010).

It is the belief of this collaborative team that the Phone Angel Program will serve as a solid model for developing productive volunteer programs among older adults in different communities and regions. Findings suggest that older Chinese are resilient and strong, and confirm that the strengths perspective approach is a best practice for developing programs with Chinese older adults. The training and regular group supervision and sharing had increased social capital and group bonding and social trust among older Chinese volunteers. Data support that the Phone Angel Program volunteers could make a positive, holistic impact on the Chinese community. The project supports the notion that older adults, regardless of background, can be educated and empowered to meet the critical needs of their own communites. Although most of the volunteers had no English language facility and have experienced difficulty participating in mainstream volunteer programs, they were still able to make a difference when professionals were willing to invest in them as human capital. The investment in older adults as human capital is worth the effort because the pay-off is priceless in terms of the older adult’s perceived improved health.

This pilot study supports that an older volunteer program can be an effective health promotion program for the older adult population. Implications for future work are (1) to replicate the Phone Angel Program in more senior centers as a health promotion option, and (2) to expand the recipient groups to other needy Chinese sub-groups including people living alone, isolated older adults, mental health patients, caregivers of special needs children and adult individuals, and new immigrants. This program appears to have the potential to provide a large segment of the older Chinese population with opportunities for meaningful productive engagement, and with improved health and mental health benefits. Future research will be needed to answer questions about the long-term impact of the Phone Angel Program on individuals and communities, strategies for older Chinese volunteer programs to be developed in different Chinese communities in other regions, and program sustainability and cost-benefit to society.

The Phone Angel Program brought cultural change to the Chinese older adult population, to the senior center and to the hospital, to their families, and to the community at large. Through the program, volunteers brought a personal investment in the welfare of fellow Chinese immigrant families and their community. Social network connections among Phone Angel Program volunteers created new avenues for information exchange and resource mobilization. The Phone Angel Program volunteers created synergies of influence across collaborating organizations and among volunteer individuals so that the organizations, and ultimately the larger community, benefited. These findings suggest that the impact of volunteers is amplified when caregiver recipients are less likely to feel isolated and unsupported.

The development and implementation of the Phone Angel Program demonstrated how a social marketing conceptual framework can serve as a social model of mental health intervention for the older Chinese population in New York City. The collaborators demonstrate how social marketing can be used to guide the development of volunteer programs for health promotion. Health prevention and promotion programs embedded in a volunteer program have the potential to engage older Chinese who might not respond to a direct appeal to improve their health and mental health, as it is appealing to their strengths, not their weaknesses.

The strength of this pilot program lay in the dual benefits for both volunteers and caregiver recipients. Using an evidence-based approach with a training curriculum, research instruments, and outcome measures ensured program replication. The program was costly in terms of social work time and expertise, but the cost was well worth the benefits: health and mental health benefits for older volunteers; provision of social support and social connection to the caregivers; and an overall benefit to society. Limitations included the small sample size, which affects generalizability of findings. However, the implementation process confirmed the feasibility of the project, the usefulness of the training curriculum, the effectiveness of partnership among social workers and organizations, and the willingness of older Chinese Americans to be volunteers.
